# Broad-Spectrum Anti-biofilm Peptide That Targets a Cellular Stress Response

**DOI:** 10.1371/journal.ppat.1004152

**Published:** 2014-05-22

**Authors:** César de la Fuente-Núñez, Fany Reffuveille, Evan F. Haney, Suzana K. Straus, Robert E. W. Hancock

**Affiliations:** 1 Department of Microbiology and Immunology, Centre for Microbial Diseases and Immunity Research, University of British Columbia, Vancouver, British Columbia, Canada; 2 Department of Chemistry, University of British Columbia, Vancouver, British Columbia, Canada; University of Washington, United States of America

## Abstract

Bacteria form multicellular communities known as biofilms that cause two thirds of all infections and demonstrate a 10 to 1000 fold increase in adaptive resistance to conventional antibiotics. Currently, there are no approved drugs that specifically target bacterial biofilms. Here we identified a potent anti-biofilm peptide 1018 that worked by blocking (p)ppGpp, an important signal in biofilm development. At concentrations that did not affect planktonic growth, peptide treatment completely prevented biofilm formation and led to the eradication of mature biofilms in representative strains of both Gram-negative and Gram-positive bacterial pathogens including *Pseudomonas aeruginosa*, *Escherichia coli*, *Acinetobacter baumannii*, *Klebsiella pneumoniae*, methicillin resistant *Staphylococcus aureus*, *Salmonella* Typhimurium and *Burkholderia cenocepacia*. Low levels of the peptide led to biofilm dispersal, while higher doses triggered biofilm cell death. We hypothesized that the peptide acted to inhibit a common stress response in target species, and that the stringent response, mediating (p)ppGpp synthesis through the enzymes RelA and SpoT, was targeted. Consistent with this, increasing (p)ppGpp synthesis by addition of serine hydroxamate or over-expression of *relA* led to reduced susceptibility to the peptide. Furthermore, *relA* and *spoT* mutations blocking production of (p)ppGpp replicated the effects of the peptide, leading to a reduction of biofilm formation in the four tested target species. Also, eliminating (p)ppGpp expression after two days of biofilm growth by removal of arabinose from a strain expressing *relA* behind an arabinose-inducible promoter, reciprocated the effect of peptide added at the same time, leading to loss of biofilm. NMR and chromatography studies showed that the peptide acted on cells to cause degradation of (p)ppGpp within 30 minutes, and *in vitro* directly interacted with ppGpp. We thus propose that 1018 targets (p)ppGpp and marks it for degradation in cells. Targeting (p)ppGpp represents a new approach against biofilm-related drug resistance.

## Author Summary

Bacteria colonize most environments, including the host by forming biofilms, which are extremely (adaptively) resistant to conventional antibiotics. Biofilms cause at least 65% of all human infections, being particularly prevalent in device-related infections, infections on body surfaces and in chronic infections. Currently there is a severe problem with antibiotic-resistant organisms, given the explosion of antibiotic resistance whereby our entire arsenal of antibiotics is gradually losing effectiveness, combined with the paucity of truly novel compounds under development or entering the clinic. Thus the even greater resistance of biofilms adds to the major concerns being expressed by physicians and medical authorities. Consequently, there is an urgent need for new strategies to treat biofilm infections and we demonstrate in the present study an approach, based on the inhibition of (p)ppGpp by a small peptide, that eradicates biofilms formed by four of the so-called ESKAPE pathogens, identified by the Infectious Diseases Society of America as the most recalcitrant and resistant organisms in our society. The strategy presented here represents a significant advance in the search for new agents that specifically target bacterial biofilms.

## Introduction

Biofilms are structured multicellular communities of microorganisms associated with surfaces. They have been widely studied, in part because they cause at least 65% of all human infections, being particularly prevalent in device-related infections, on body surfaces (skin and soft tissue, lung, bladder, endocarditis, etc.) and in chronic infections [1], [2]. They represent a major health problem worldwide due to their resistance to host defence mechanisms and to conventional antimicrobials, which generally target free-swimming (planktonic) bacteria [1], [2]. Hence, there is an urgent need to identify compounds that effectively clear biofilm-related infections.

Bacteria are known to respond to stressful environmental conditions (such as starvation) by activating the stringent response (SR) [3]. As a consequence, the cell synthesizes two small signaling nucleotides, guanosine 5′-diphosphate 3′-diphosphate (ppGpp) and guanosine 5′-triphosphate 3′-diphosphate (pppGpp), collectively denoted (p)ppGpp [3]. These serve as a second messenger response that is induced by a variety of stress conditions, is highly conserved in both Gram-negative and Gram-positive species [3], [4], regulates the expression of a plethora of genes [3], and is known to play a role in biofilm formation in certain species [5]–[11], although some variability has been observed [6], [7], [9], [12].

Synthetic cationic peptides, derived from natural peptides such as the human cathelicidin LL-37 and the bovine peptide indolicidin [13], have been recently identified as biofilm inhibitory compounds [14]. Anti-biofilm peptides are similar to cationic antimicrobial peptides (which are active against planktonic bacteria), comprising both cationic and hydrophobic amino acids [14], but have substantially different structure-activity relationships. Thus, we previously identified peptides with good anti-biofilm but virtually no activity vs. planktonic bacteria (i.e., very high MIC values) [14], and vice versa. Moreover, certain anti-biofilm peptides are active against biofilms formed by *Burkholderia cenocepacia* [14], a pathogen that is completely resistant to all antimicrobial peptides in the planktonic state. The broad-spectrum activity of anti-biofilm peptides [14] suggests that they target a biofilm-specific process common amongst bacteria. Given the above, we hypothesized that anti-biofilm peptides exerted their activity by blocking a widespread stress response that contributes to biofilm development, and that this was indeed the stringent response mediated through (p)ppGpp. Here, we have identified a peptide that has very broad spectrum activity against many of the most antibiotic-resistant species of concern in human medicine and provide evidence it acts to promote (p)ppGpp degradation.

## Results

### Peptide 1018 as a potent broad-spectrum anti-biofilm agent

**Figure 1:**
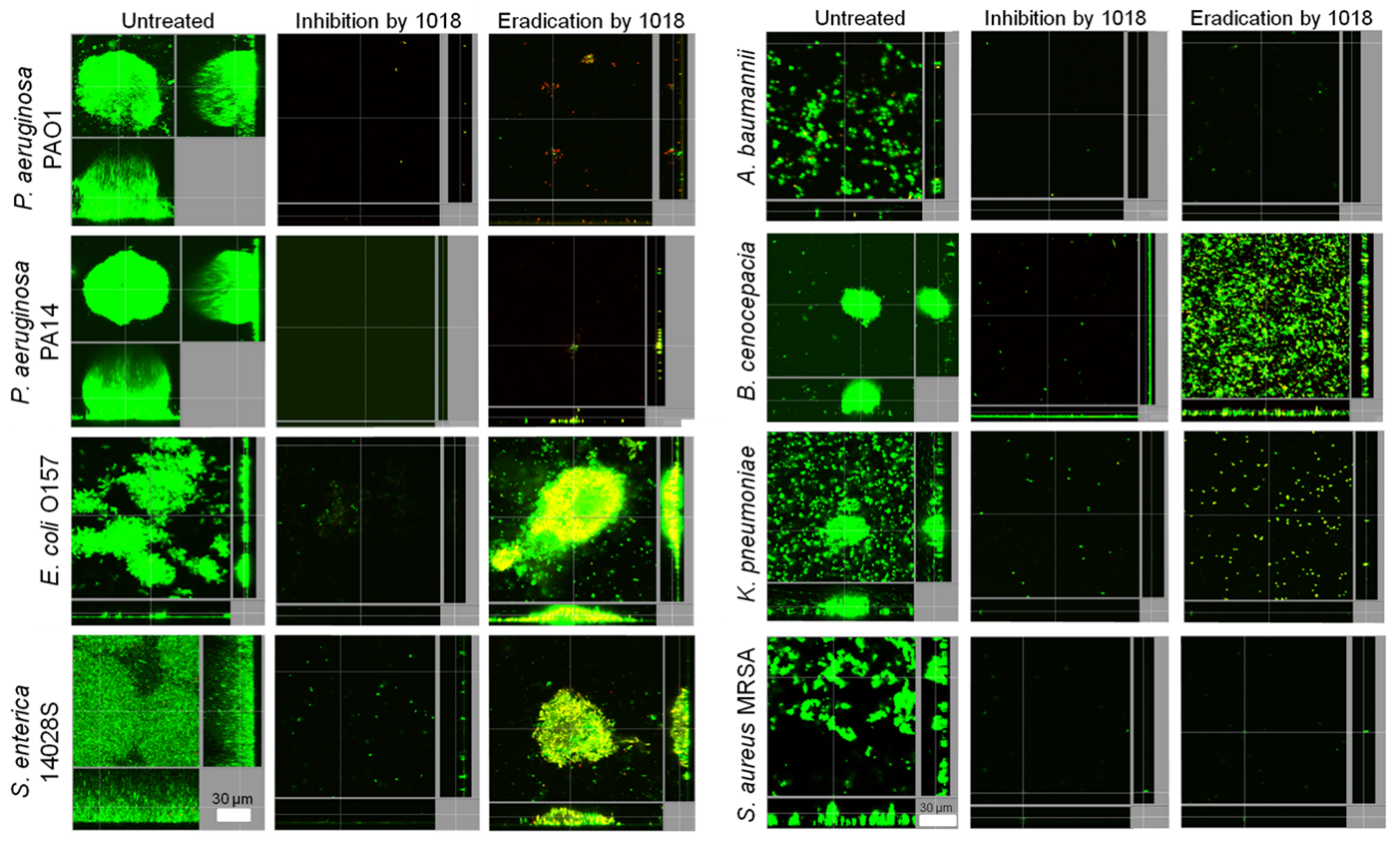
Peptide 1018 potently inhibited bacterial biofilms at concentrations that did not affect planktonic cell growth. Sub-inhibitory concentrations of peptide 1018 prevented biofilm development and eradicated or reduced existing biofilms of Gram-negative and Gram-positive bacteria. Concentrations of peptide 1018 used were 10 µg/ml for *Pseudomonas aeruginosa* (labelled as strains PA14 and PAO1) *Escherichia coli* 0157, *Acinetobacter baumannii* and *Burkholderia cenocepacia*, 20 µg/ml for *Salmonella enterica* serovar Typhimurium 14028S experiments, 2 µg/ml for *Klebsiella pneumoniae* experiments, and 2.5 µg/ml for methicillin resistant *Staphylococcus aureus* (MRSA) experiments. Inhibition of biofilm development was tested by immediately adding 1018 into the flow-through medium of the flow cell apparatus and then monitoring biofilm formation for 3 days. Eradication conditions involved waiting two days before addition of 1018 into the flow-through medium. After 3 days, bacteria were stained green with the all bacteria stain Syto-9 and red with the dead-bacteria stain propidium iodide (merge shows as yellow to red) prior to confocal imaging. Each panel shows reconstructions from the top in the large panel and sides in the right and bottom panels (xy, yz and xz dimensions).

While screening for peptides with anti-biofilm activity, we identified the previously unknown ability of the immunomodulatory peptide IDR (innate defense regulator)-1018 (VRLIVAVRIWRR-NH_2_; abbreviated here as 1018) [15] to specifically target and kill biofilm cells (Fig. 1), at much lower concentrations than previously described peptides [14]. At concentrations that had no effect on planktonic growth (Table 1), this peptide was able to potently prevent biofilm formation (Fig. 1, middle panels) and eradicate preformed (2-day old) biofilms (Fig. 1, right hand panels) formed by diverse species of Gram-negative bacteria and the Gram-positive bacterium *Staphylococcus aureus*.

**Table 1:** Peptide 1018 exhibited potent broad-spectrum direct anti-biofilm activity but weak antibacterial activity for planktonic cells.

Bacterial strains	MIC (µg/ml)	MBIC_50_ (µg/ml)	MBIC_100_ (µg/ml)
*Pseudomonas aeruginosa* PA01	64	5	10
*Pseudomonas aeruginosa* PA14	64	5	10
*Burkholderia cenocepacia* IIIa 4813	>256	2	10
*Escherichia coli* 0157	32	8	10
*Acinetobacter baumannii* SENTRY C8	128	2	10
*Klebsiella pneumoniae* ATTC13883	8	2	2
*Salmonella enterica* sv Typhimurium 14028S	64	3.2	10
*S. aureus* MRSA #SAP0017	64	2	2.5

Comparison of planktonic cell MIC to MBIC_50_ and MBIC_100_, which are the minimal biofilm inhibitory concentrations leading to 50% and 100% decrease in biofilm growth, respectively.

**Figure 2:**
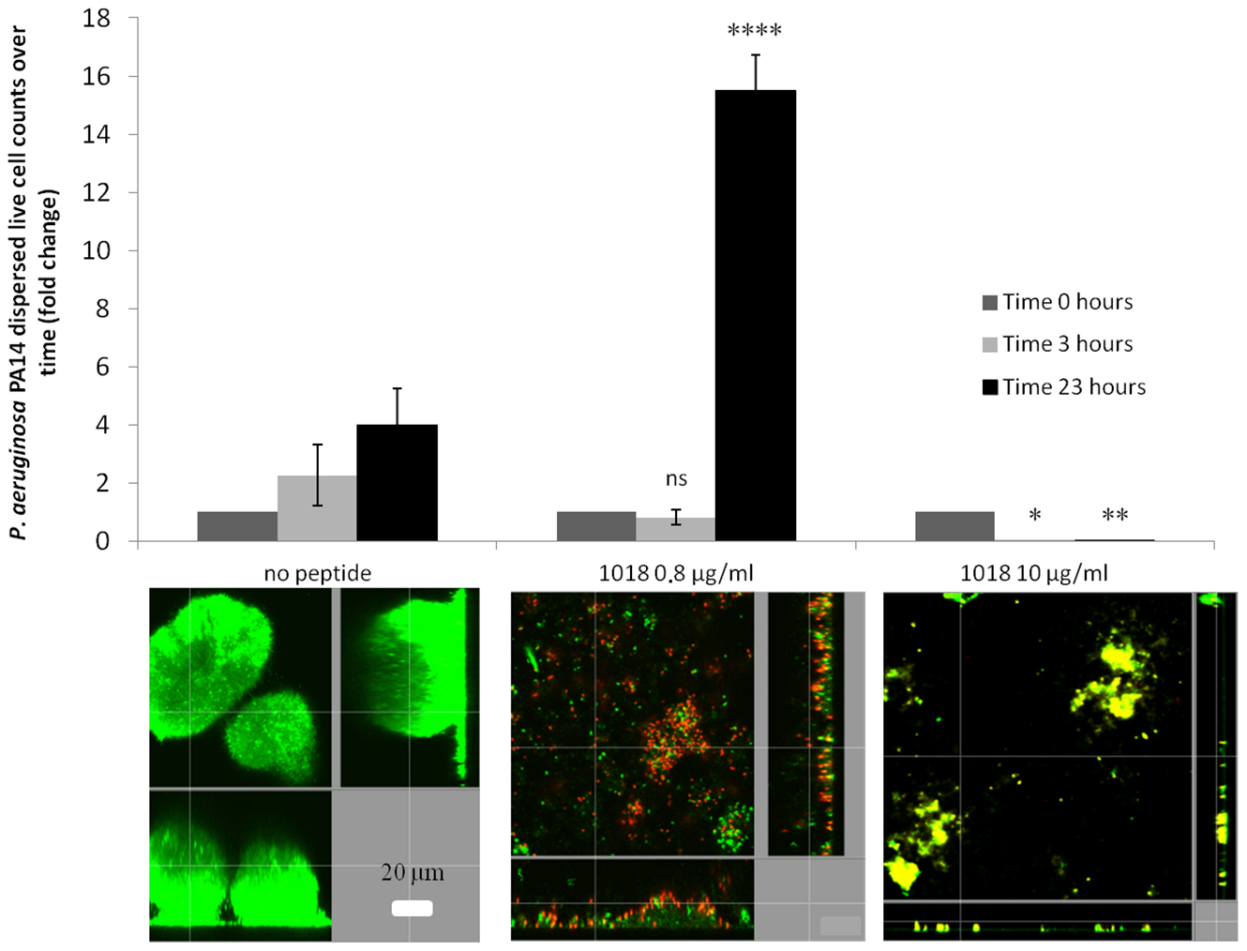
Low levels of 1018 led to biofilm dispersion while higher levels triggered biofilm cell death. Dispersed cells from mature *P. aeruginosa* flow cell biofilms were collected and viable cell counts performed after 0, 3 and 23 h of treatment with different concentrations of the peptide (0.8 and 10 µg/ml). Representative confocal images of the remaining cells present in the flow cell chambers after peptide treatment are shown for each condition. Statistical significance comparing peptide-treated groups to untreated was determined using Student's *t* test (ns, P>0.05; *, P<0.05; **, P<0.01; ****, P<0.0001).

We investigated the role of 1018 in biofilm cell dispersion and killing of *P. aeruginosa* PA14 2-day old biofilms. At very low concentrations (0.8 µg/ml), the peptide increased live cell dispersion from existing biofilms by ∼4-fold after 23 h of treatment (Fig. 2), resulting in an average of 8.2±6.6% residual biofilm biovolume compared to the untreated controls (P<0.05). Only 26±7.4% of the cells that remained attached within the flow cell chambers were killed by treatment with 0.8 µg/ml 1018. Conversely, higher concentrations of peptide (10 µg/ml) did not trigger live biofilm cell dispersal (Fig. 2), and most of the cells remaining bound to the surface were dead, as judged by uptake of the normally impermeant stain propidium iodide (67±7.7% red cells compared to 2.5±1.0% in the untreated controls; P<0.05).

In experiments with pre-grown, 2-day old biofilms, only *E. coli* 0157, *S. enterica* 14028S and *B. cenocepacia* samples consistently had attached cells after peptide treatment (Fig. 1, right hand panels). However, the remaining cell population was mostly dead in the case of *E. coli* 0157 as, on average, there was a significant increase in dead cell number in treated samples (54.4±3.1%) compared to untreated samples (1.5±0.9%; P<0.05). On the other hand, the peptide caused more substantial dispersal but lesser cell death in *S. enterica* 14028S and *B. cenocepacia* biofilms. *Salmonella* biofilms treated with 1018 had 29.2±19.0% dead cells as opposed to just 0.54±0.69% in samples without peptide (P<0.05). *B. cenocepacia* biofilms exhibited no significant increase in cell death (7.9±3.4% cf. 3.8±2.4% in the untreated controls).

### Evidence for a role for the stringent response

The basis for the broad-spectrum activity of peptide 1018 was investigated. Previous studies, based on transcriptomic and biochemical investigations, have suggested that peptides LL-37 [13] and 1037 [14] act against *Pseudomonas* by modestly inhibiting attachment and quorum sensing as well as promoting twitching motility. However, although we could show that 1018 had similar modest effects on these processes, it was difficult to rationalize these mechanisms with the observed broad-spectrum activity, since these processes vary substantially within the above-described target species. Thus we considered that there might be a common mechanism and hypothesized that the peptide acted to inhibit a common stress response in target species, namely the so-called stringent response, mediating (p)ppGpp synthesis through the enzymes RelA and SpoT.

**Figure 3:**
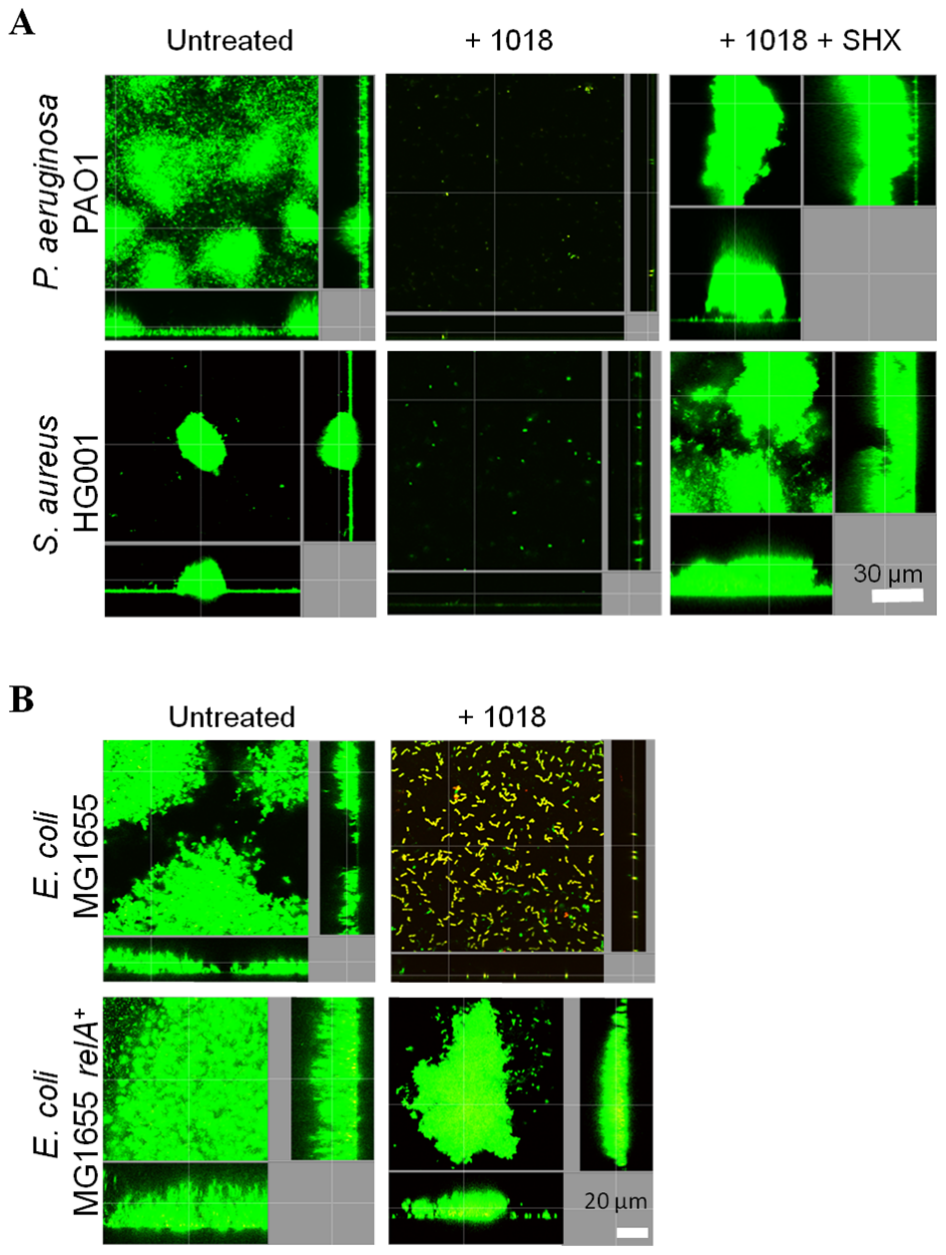
Enhanced (p)ppGpp production leads to altered susceptibility of biofilms to peptides. (**A**) Addition of SHX, which leads to overproduction of (p)ppGpp, resulted in the resistance of biofilm development to 20 µg/ml of peptide 1018; (**B**) (p)ppGpp overproduction through *relA* overexpression led to anti-biofilm peptide resistance. (**A–B**) Inhibition of biofilm development was tested by immediately adding 20 µg/ml 1018 (± SHX or IPTG) into the flow-through medium of the flow cell apparatus and then monitoring biofilm formation for 3 days. After 3 days, bacteria were stained green with the all bacteria stain Syto-9 prior to confocal imaging. Each panel shows reconstructions from the top in the large panel and sides in the right and bottom panels (xy, yz and xz dimensions).

Overproducing the potential target of a given drug is a well-established method for identifying drug targets. Here we overproduced (p)ppGpp by addition of serine hydroxamate (SHX; a structural analogue of L-serine that induces the stringent response by inhibiting charging of seryl-tRNA synthetase [16]), and by IPTG induction of the cloned *relA* gene, and observed resistance against peptide 1018 (Fig. 3A,B). First we performed checkerboard microtiter plate assays, using established methods [17], to analyze the interaction between SHX addition at time 0 and 1018 treatment in more detail. Minor modifications were made to previously described methodology [17] to quantify adherent biofilm biomass (as opposed to planktonic bacterial growth) using the crystal violet assay [14]. The crystal violet-stained biofilm was resuspended using 70% ethanol and quantified using a spectrophotometer at 595 nm. Three independent experiments were performed and statistical significance was determined using Student's *t* test. At concentrations of SHX (10 µM) that did not affect *P. aeruginosa* PAO1 planktonic growth (which required 250 µM SHX to inhibit growth, Fig. S1C), we observed increased biofilm formation by nearly 2-fold (to 188±0.3% cf. the SHX untreated control; P<0.05). In these cells the minimal biofilm inhibitory concentration (MBIC) went from 10 µg/ml (Table 1) to 80 µg/ml of 1018 (leading to a reduction to 8.9±0.02% biofilm volume cf. the peptide untreated control; P<0.05; no difference was observed at 40 µg/ml of the peptide, which led to 93.2±0.04% biofilm formation cf. the peptide untreated control). These results clearly showed that peptide resistance was not due to slow growth. At 320 µM SHX, whereby biofilm production was increased nearly 4-fold (to 395±0.4% cf. the SHX untreated control; P<0.01), 160 µg/ml 1018 was required to fully inhibit biofilm formation (reduced to 4.7±0.002% cf. peptide untreated control; P<0.01). Thus the amount of peptide required to inhibit biofilms depended on the concentration of SHX, and therefore on the levels of (p)ppGpp, since increasing the levels of SHX resulted in peptide resistance unless a higher dose of peptide was used. While SHX by itself clearly resulted in an increase in biofilm development over the 18 to 24 h of the assay, the peptide was present in these studies even before biofilms began to develop.

These results were confirmed and extended using flow cell methods. Overproduction of (p)ppGpp by SHX treatment of *P. aeruginosa* and *S. aureus* wild-type strains led to peptide resistance (Fig. 3A). The fold-change in biovolume of *P. aeruginosa* PAO1 biofilms treated with 20 µg/ml 1018 was 0.095±0.03 (P<0.05) compared to untreated controls. However, adding SHX restored the ability to form biofilms in the presence of the peptide (2.6±0.5 fold-increase compared to untreated samples; P<0.05). Similarly, the biovolume of 1018-treated *S. aureus* HG001 biofilms was only 0.6% that of untreated samples, which was complemented when adding SHX (1.65±0.6 fold-change compared to untreated samples; P<0.05). Similar results were obtained by genetic means whereby peptide resistance was increased by overproduction of (p)ppGpp in an *E. coli* strain overexpressing the cloned *relA* gene under the control of an IPTG-inducible promoter (Fig. 3B).

### Evidence for (p)ppGpp as an important signal in biofilm growth

**Figure 4:**
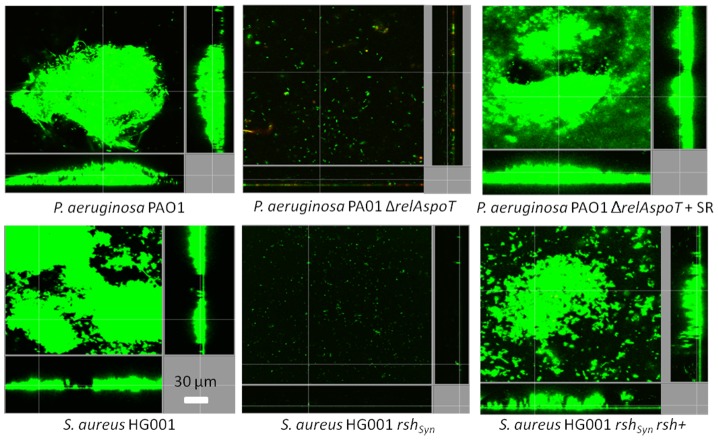
Genetic complementation of (p)ppGpp synthetase enzymes restored the ability to form biofilms. The biofilm deficiency of *Pseudomonas aeruginosa* PAO1 and *Staphylococcus aureus* HG001 (p)ppGpp mutants (Δ*relAspoT* and *rsh_Syn_* respectively) was rescued by genetic complementation [Δ*relAspoT* + *relAspoT^+^* (+SR) as described [12] and *rsh^+^* [30], respectively] leading to biofilm formation equivalent to WT shown in the left-most panels. After 3 days, bacteria were stained green with the all bacteria stain Syto-9 and red with the dead-bacteria stain propidium iodide (merge shows as yellow to red) prior to confocal imaging. Each panel shows reconstructions from the top in the large panel and sides in the right and bottom panels (xy, yz and xz dimensions).

Previous studies have reported that mutants influencing (p)ppGpp production are biofilm-deficient but not always completely defective, occasionally forming monolayers of attached cells or extremely-deficient biofilms (as opposed to well-structured biofilms) [7]–[11]. To confirm that there was a correlation between the production of (p)ppGpp and biofilm production under the experimental conditions reported here, biofilm formation of (p)ppGpp-deficient mutants was compared to their respective wild-type strains in Gram-negative *Pseudomonas aeruginosa*, *Salmonella enterica* serovar Typhimurium, and *Escherichia coli* and the Gram-positive bacterium *Staphylococcus aureus* (Fig. 4, Fig. S1A). Cells unable to synthesize (p)ppGpp showed a substantial decrease in their ability to adhere tightly to the plastic surface of flow cell chambers and were unable to develop structured biofilms, although they formed residual aggregates (Fig. 4, Fig. S1A). Genetic complementation of the genes responsible for (p)ppGpp synthesis in *P. aeruginosa relA spoT* and *S. aureus rsh* mutants restored the full ability to form biofilms (Fig. 4). In the un-complemented mutants (Fig. 4, Fig. S1A), residual (p)ppGpp-deficient mutant cells appeared to be in the planktonic state as opposed to adhering to the surface, and often were dead or division-inhibited (demonstrating filaments) (Fig. S1B). These poorly-attached cells could be cleared by increasing the flow rate. This might explain in part the variability in the defect in biofilm formation in mutants defective in (p)ppGpp production (i.e. due to flow rate, or other factors such as the age of the biofilms, temperature and media utilized here, which differed compared to previous reports; 6,7,9,12).

**Figure 5:**
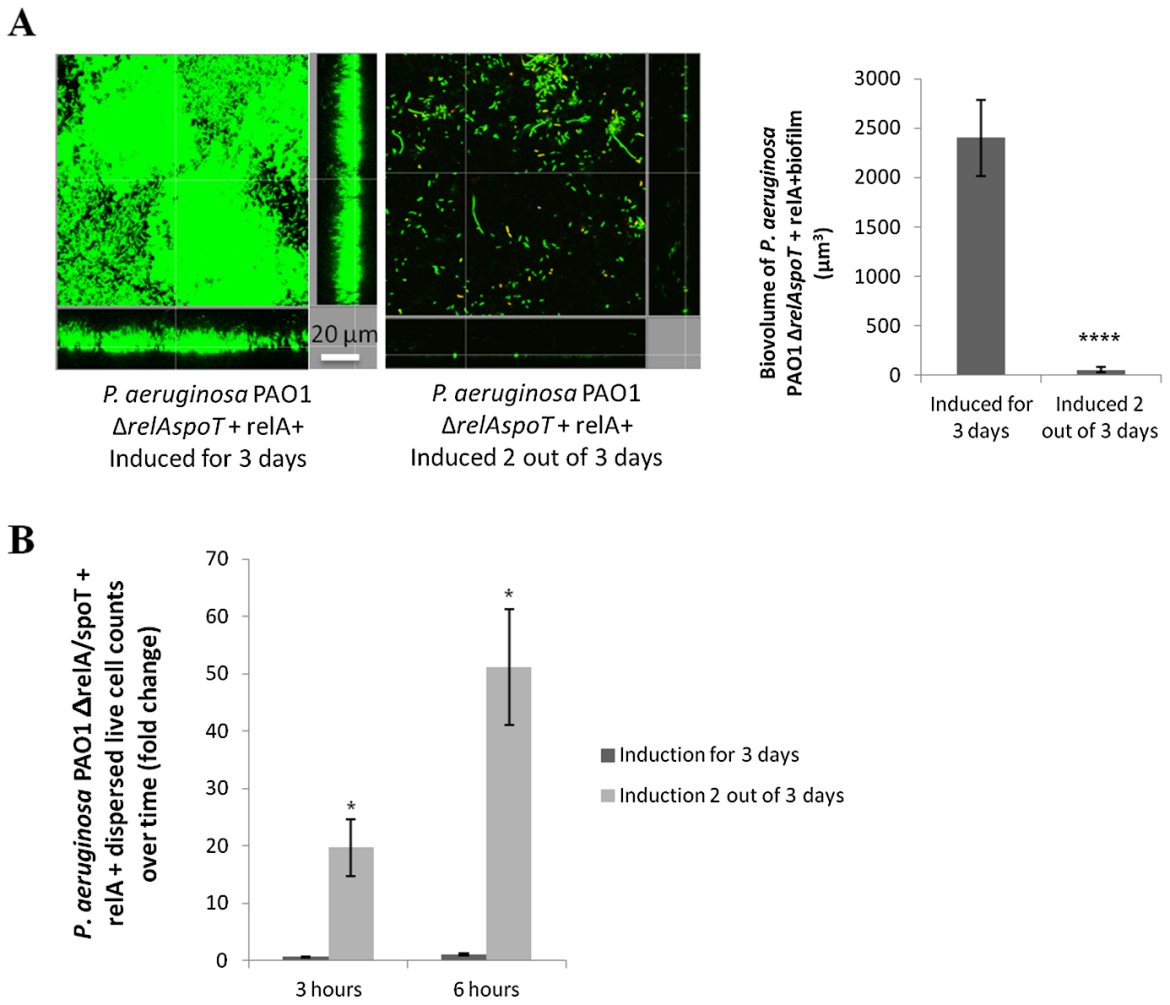
Modulation of *relA* expression impacts on biofilm development. (**A**) ***relA*** **expression modulated biofilm formation and disassembly.** The *relA* gene under the control of an arabinose-inducible promoter was introduced into a *P. aeruginosa* PAO1 Δ*relAspoT* background. Induction of *relA* led to biofilm formation in flow cells after 3 days. On the other hand, induction of *relA* for 2 days followed by 24 h of non-induction led to biofilm dispersal. Biofilm biovolume was calculated using Imaris software from Bitplane AG. Experiments were performed at least in triplicate. Student's *t* test was used (****, P<0.0001). (**B**) Repression of *relA* expression (after 2 days of induction) led to biofilm dispersal in a *P. aeruginosa* PAO1 Δ*relAspoT* strain, while continuous induction of *relA* expression during the 3 days of the experiment resulted in significantly fewer cells dispersed from biofilms. Dispersed cells from 2-day old biofilms were collected and viable cell counts performed 3 and 6 h after induction of *relA* expression was either stopped or continued. Statistical significance was determined using Student's *t* test (*, P<0.05).

To further demonstrate the role of (p)ppGpp in biofilm development and maintenance, we introduced the *relA* gene under the control of an *araC* promoter into *P. aeruginosa* PAO1Δ*relAspoT* such that it expressed *relA* upon arabinose induction. Biofilms of this strain that were expressing *relA* due to the introduction of arabinose into the flow medium during the 3-day experiment, were able to form well-structured biofilms (Fig. 5A). However, when induction of *relA* was stopped at day 2 (i.e. for the last 24 h of the experiment by removal of arabinose from the flow medium), analogous to delayed treatment by peptide 1018, pre-formed biofilms were dispersed (Fig. 5A). Indeed, we performed viable cell counts of dispersed cells from these biofilms and found that repressing *relA* expression after 2 days of continuous induction led to biofilm dispersion (Fig. 5B), while continued induction of *relA* for the 3 days of the experiment resulted in significantly reduced dispersal levels (Fig. 5B) that were similar to that of the wild-type strain (data not shown). These results clearly highlighted the roles that *relA*-dependent (p)ppGpp production play both in biofilm formation and in biofilm maintenance, as well as the consequences of blocking (p)ppGpp synthesis.

### Involvement of (p)ppGpp in 1018 action

**Figure 6:**
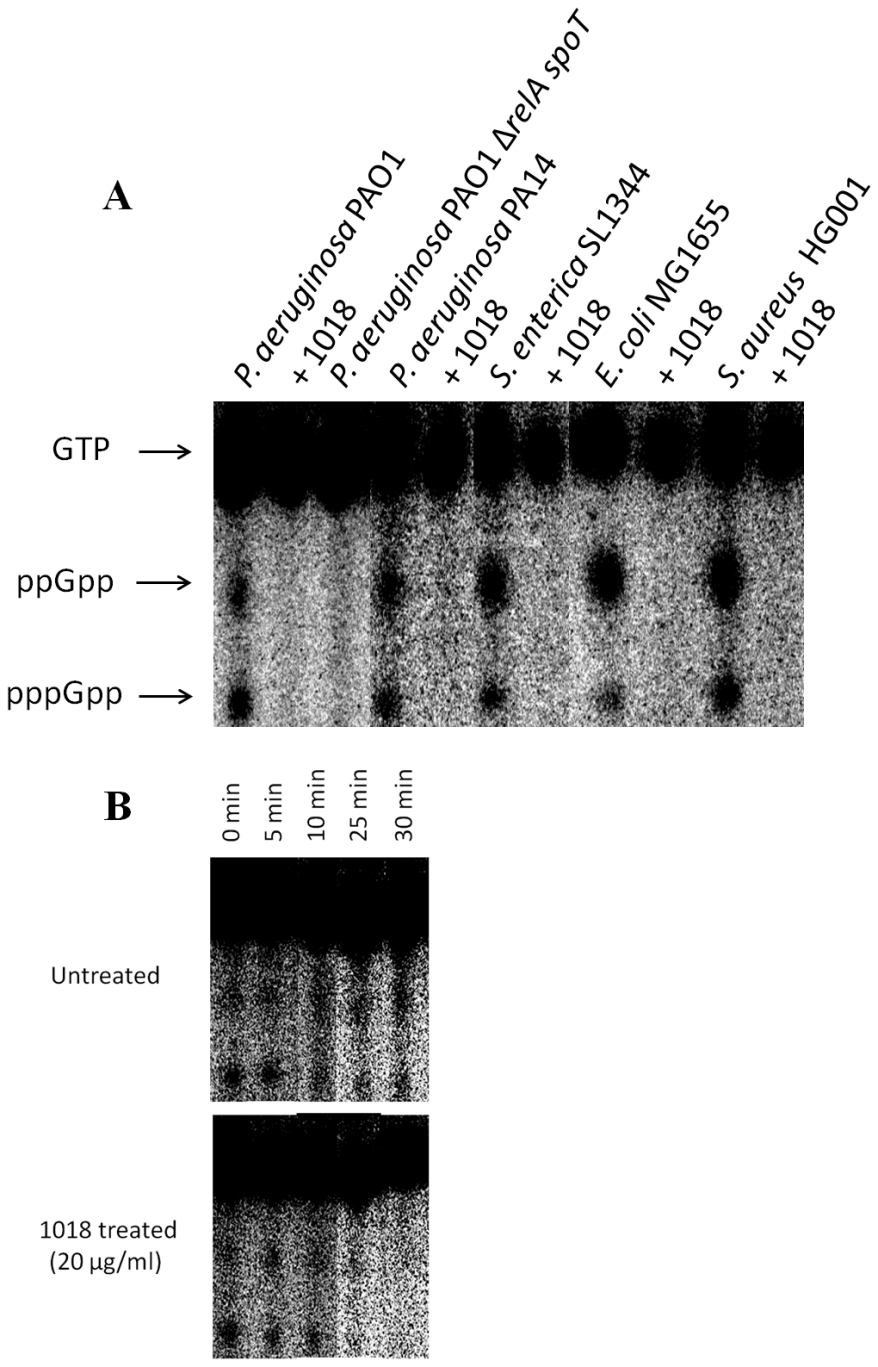
Peptide 1018 prevented (p)ppGpp accumulation *in vivo* as revealed by thin layer chromatography separation of guanine nucleotides extracted from intact cells. (**A**) Anti-biofilm peptide 1018 at 5 µg/ml directly prevented (p)ppGpp accumulation. (**B**) Treatment with peptide 1018 led to (p)ppGpp elimination within 30 min in *P. aeruginosa* PAO1 cells containing pre-accumulated (p)ppGpp due to SHX treatment. In panel **A**, bacteria were grown overnight in modified MOPS minimal medium containing 0.4% glucose, 2 mM phosphate (KH_2_PO_4_), and 0.2% CAA. For experiments evaluating the ability of the peptide to directly degrade (p)ppGpp in panel **B**, the cells were grown as described previously, induced with SHX and allowed to synthesize (p)ppGpp for 3 h prior to peptide treatment. After growth for both A and B, the cells were then diluted 1∶20 in the same MOPS minimal medium except containing 0.4 mM phosphate (KH_2_PO_4_) and 500 µM serine hydroxamate (SHX) to induce (p)ppGpp synthesis, in the presence or absence of peptide 1018 and cells were labelled with 10 µCi/ml ^32^P for 3 h. Samples were then extracted with frozen 13 M formic acid by three cycles of freeze-thaw. Aliquots of the supernatants were applied to 20×20 cm PEI cellulose TLC plates, resolved with 1.5 M KH_2_PO_4_, pH 3.4 for 4 h. After chromatography, nucleotides were visualized by autoradiography and quantified with a MolecularImager FX PhosphorImager and Quantity One software (Bio-Rad). Controls were performed to demonstrate that the Δ*relAspoT* mutation also prevented (p)ppGpp formation.

The role of (p)ppGpp in the anti-biofilm mechanism of peptide 1018 was further assessed in multiple species. Direct measurement of the cellular levels of (p)ppGpp by thin layer chromatography (TLC) revealed that cells from multiple bacterial species treated with 5 µg/ml of peptide 1018 did not accumulate (p)ppGpp (Fig. 6A and Fig. S4B). In contrast, the conventional cationic antibiotics colistin, polymyxin B and tobramycin were unable to prevent (p)ppGpp accumulation (Fig. S3, left panel; indeed the latter two actually increased ppGpp) or cause degradation of accumulated (p)ppGpp (Fig. S3, right panel), thus demonstrating that these cationic antibiotics did not utilize a similar mechanism to that of peptide 1018.

**Figure 7:**
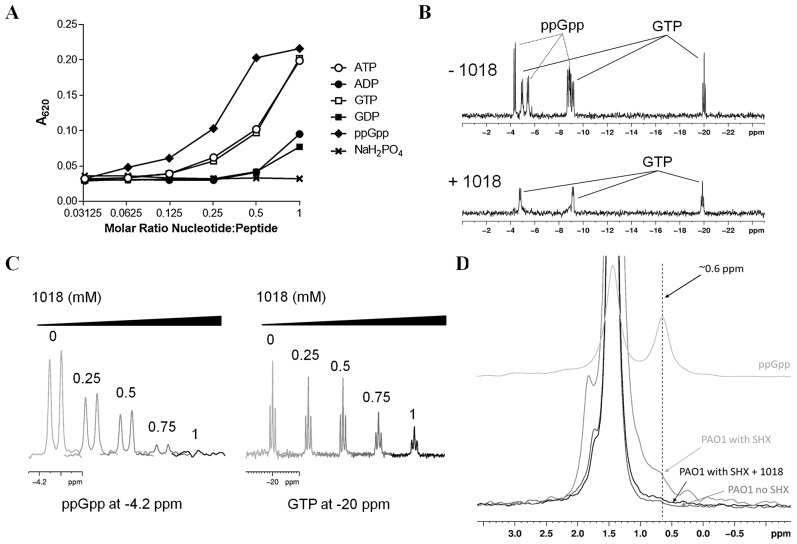
Peptide 1018 bound to ppGpp *in vitro* and led to degradation of (p)ppGpp *in vivo*. (**A**) Binding of peptide 1018 to various nucleotides based on co-precipitation. Peptide 1018 (0.25 mM) was separately mixed with increasing amounts of ppGpp, GTP, ATP, GDP and ADP in buffer (50 mM Tris, pH 7.4) and the extent of co-precipitation was assessed by measuring the increase in absorbance at 620 nm. The amount of co-precipitation induced by 1018 appeared to correlate with an increased negative charge on the nucleotides. A separate sample containing NaH_2_PO_4_ revealed that phosphate ions did not induce precipitation of 1018 in the concentration range tested. (**B**) Anti-biofilm peptide 1018 preferentially bound to ppGpp compared to GTP as revealed by ^31^P-NMR spectroscopy. In the absence of 1018 (top panel), a mixture of 0.5 mM ppGpp and 0.5 mM GTP revealed unique signals corresponding to the phosphorous atoms in ppGpp and GTP (indicated by arrows). Upon the addition of 1 mM 1018 (bottom panel), the peak intensity from the ppGpp signals was almost completely abolished, while the signals from GTP were reduced but to a lesser degree. (**C**) Samples containing an equimolar mixture of ppGpp and GTP at intermediate concentrations of 1018 were used to further evaluate the preferential binding of 1018 to ppGpp. Examination of specific spectral regions unique to ^31^P signals from either ppGpp (∼−4.2 ppm) or GTP (∼−20 ppm) showed that the ppGpp peak intensity decreased more readily than those from GTP (peptide concentrations, in mM, are indicated above each trace). The preferential precipitation of ppGpp by 1018 suggests that the peptide had a higher affinity for ppGpp over GTP under these conditions (See also Fig. S5B). (**D**) The ppGpp levels in nucleotide extracts from *P. aeruginosa* PAO1 cultures induced with SHX and treated with 1018 were also measured using ^31^P-NMR spectroscopy. In these spectra, the ppGpp phosphorous signals were shifted because of the presence of 6.5 M formic acid used to extract the nucleotides from the PAO1 cells. The chemical shifts of GTP and ppGpp in 6.5 M formic acid were determined separately using samples of pure nucleotide (See Fig. S6). Only the region from 3 ppm to −0.5 ppm is shown as this contained a unique ppGpp phosphorous peak at 0.6 ppm (for comparison, the spectra of 0.5 mM ppGpp in 6.5 M formic acid is shown as the top trace). In the ^31^P spectrum of nucleotide extracts from PAO1 induced with SHX, the ppGpp peak at 0.6 ppm appeared as a shoulder on the large phosphate peak at 1.5 ppm (middle grey trace). This shoulder was absent in samples taken from PAO1 cells grown without SHX (lowest grey trace). When PAO1 induced with SHX was treated with 20 µg/ml 1018, the ppGpp peak was essentially lost (black trace) demonstrating that the addition of 1018 to bacteria leads to the degradation of ppGpp *in vivo*.

The peptide was able to interact directly with ppGpp as demonstrated by co-precipitation (Fig. 7A) and TLC of residual ppGpp (Fig. 6, Fig. S4B) and by nuclear magnetic resonance spectrometry (NMR) of the complexed molecules (Fig. 7B–D). These studies further showed that peptide 1018 preferentially bound to ppGpp compared to other nucleotides such as GTP (Fig. 7A, Fig. S5B).

We then investigated the mechanism by which the peptide 1018-ppGpp interaction led to the loss of the ppGpp signal in cells. One possibility was that the peptide sequestered the nucleotide, forming a peptide-ppGpp complex, which prevented ppGpp detection in our TLC and NMR assays. However, we observed that formic acid (used to extract nucleotides in both TLC and NMR experiments) led to a disruption of the peptide-ppGpp complex while maintaining ppGpp in its intact form (Fig. S5A, right panel), so this explanation seems unlikely as no ppGpp was visible on TLC and NMR after formic acid treatment (Fig. 6, Fig. 7D, Fig. S5A). Alternatively, peptide treatment could lead to ppGpp degradation. In agreement with this second possibility, TLC and NMR analysis of *in vivo* experiments showed that the addition of peptide led to the rapid degradation of (p)ppGpp within cells that had pre-accumulated these nucleotides (Fig. 6B, Fig. 7D). In the TLC experiments, (p)ppGpp synthesis was induced in *P. aeruginosa* PAO1 cultures by SHX for 3 h, after which 20 µg/ml of the peptide was added and the fate of (p)ppGpp was monitored over time, revealing elimination within only 30 min (Fig. 6B). Likewise, after treatment with SHX to induce (p)ppGpp synthesis in *P. aeruginosa* PAO1 cells and then treatment with peptide for 1 h followed by extraction of nucleotides and NMR, the ppGpp peak observed in untreated cells was dramatically reduced (Fig. 7D). Taken together, these results indicate that peptide 1018 directly and specifically interacts with (p)ppGpp and triggers its degradation, thus preventing its signaling effects within the cell (e.g. its role in biofilm development and maintenance).

## Discussion

The results described here indicate that an anti-biofilm peptide can exhibit potent broad-spectrum activity due to its ability to depress (p)ppGpp levels in live bacterial cells. Previous studies have suggested that the stringent response might be involved in biofilm formation [5]–[11], although some controversy exists as to whether this may be due to the particular experimental conditions used, particularly flow rate and age of the biofilms examined. The stringent response is induced in reaction to bacterial stresses such as amino-acid starvation, fatty acid, iron or nutritional limitation, heat shock, and other stress conditions. It is signaled by the alarmone (p)ppGpp, and modulates transcription of up to one third of all genes in the cell. Conceptually it is designed to divert resources away from growth and division and toward metabolism in order to promote survival. Its specific role in biofilm formation is not known but it may be involved in initiating and/or perpetuating biofilm development. In addition, the results presented here indicate that it may actually suppress the tendency of biofilms to disperse (Fig. 5), and even promote viability in adhered cells (Fig. S1B). Consistent with its effects on biofilms, *in vivo* studies have shown that (p)ppGpp-deficient mutants are easily cleared by the host, unable to establish chronic infections, incapable of long-term survival and overall more susceptible to exogenous stresses than their parent strains [18], [19]. Interestingly, various classes of antibiotics are known to induce (p)ppGpp synthesis [20-23; Fig. S3], which in turn leads to antibiotic adaptive resistance [12], [24]. Importantly, recent efforts have identified molecules that target the stringent response [25].

Here we have demonstrated that peptide 1018, which triggers the degradation of the (p)ppGpp signal within the cell, acts as a broad-spectrum biofilm inhibitor. Given the importance of biofilms in human medicine, constituting at least 65% of all infections, and increasing antibiotic resistance which in biofilms is adaptive and broad spectrum, such a peptide offers considerable potential in the fight against the burgeoning resistance to antibiotics. Peptides with biofilm inhibitory activities have been previously identified [13], [14], [26]. However, the mechanism of action by which these peptides selectively target and kill biofilm cells of both Gram-negative and Gram-positive bacteria was previously postulated to involve changes in motility, adherence and quorum sensing [13], [14], which are all species-specific and thus do not satisfactorily explain the action of peptide 1018 against a broad range of pathogens. Here we provide evidence that an anti-biofilm peptide, 1018, potently inhibited biofilm formation and eradicated existing mature biofilms in a broad-spectrum manner, through a direct interaction with ppGpp, which led to its degradation in live bacterial cells. The mechanism of action involves direct contact between peptide 1018 and (p)ppGpp so the peptide must be able to cross the cell membranes to reach the cytoplasm. Previous studies have demonstrated that amphipathic cationic peptides, like 1018, have the characteristics of so-called cell penetrating peptides that are able to freely translocate across membranes [27].

The peptide had at least three effects on biofilms, which might reflect the role of (p)ppGpp in cells. First when added prior to initiation of biofilms it prevented biofilm formation, second it specifically led to cell death in biofilms at concentrations that were not lethal for planktonic (free-swimming) cells (Table 1; Figs. 1 and 2), and third it promoted biofilm dispersal even in maturing (2-day old) biofilms (Fig. 2), effects that were in fact reciprocated in mutants unable to accumulate (p)ppGpp (Fig. 5). We suggest that the ability to cause cell death in biofilms might have been due to inhibition of cell wall biosynthesis and triggering of murine hydrolases, a known tendency for antimicrobial peptides [28]. Critically we propose that this is due in part to the impact of the stringent response in bacteria growing in the biofilm state, since stringent response is known to influence susceptibility to cell wall specific antibiotics, presumably through effects on cell wall synthesis [24], while the lack of ppGpp leads to cell death through the Slt soluble lytic transglycosylase [29]. Consistent with this, both peptide-treated biofilms and (p)ppGpp deficient mutants grown in flow cells demonstrated increased bacterial cell filamentation (Fig. S1B) and cell lysis/death (Figs. 1, 2, S1B and Fig. 5).

Previous studies have demonstrated that the structure-activity relationships of anti-biofilm peptides vary substantially from antimicrobial peptides [14] despite certain common features (being amphipathic molecules with excess cationic and hydrophobic amino acids). Consistent with this, 1018 was able to potently inhibit *Burkholderia cenocepacia* biofilms, despite the fact that this species is completely resistant to all antimicrobial peptides. Thus we have an opportunity to now develop more active peptides that have even more potent anti-biofilm activity. Indeed we have recently started to isolate such peptides and obtained preliminary evidence that they also act by inhibiting the stringent response.

## Materials and Methods

### Bacterial strains

Strains utilized included wild-type strains of *Pseudomonas aeruginosa* PAO1, strain H103, and PA14 and clinical isolates *E. coli* O157, *Salmonella enterica* serovar Typhimurium (clinical isolate 14028S), *Staphylococcus aureus* MRSA (clinical isolate #SAP0017), *Klebsiella pneumoniae* ATTC 13883 (a colistin-heteroresistant reference strain from American Type Culture Collection, Rockville, MD), *Acinetobacter baumannii* SENTRY C8 (a polymyxin B resistant blood clinical isolate from the U.S.A. obtained through the SENTRY surveillance system) and *Burkholderia cenocepacia* genomovar IIIa (Vancouver Children's Hospital clinical isolate 4813). *P. aeruginosa* PAO1 (p)ppGpp mutant Δ*relAspoT* [(Δ*relA* (Δ181-2019) Δ*spoT* (Δ200-1948)] and its complemented strain Δ*relAspoT*+SR were a kind gift from D. Nguyen [12]. *S. aureus* parent strain HG001, its (p)ppGpp mutant HG001 *rsh*_syn_ (Δ942–950 nt) and the strain complemented with full length *rsh* were kindly provided by T. Geiger [30]. *S. enterica* serovar Typhimurium parent strain SL1344 and its (p)ppGpp mutant SL1344 Δ*relAspoT* (Δ*relA*71::kan *rpsL* Δ*spoT*281::cat) were provided by K. Tedin [31]. *Escherichia coli* parent strain (MG1655), *E. coli* Δ*relAspoT* [Δ*relA*:: kan (Δ209-2302) Δ*spoT*:: cat (Δ700-2355)] deletion insertion mutant and *E. coli relA*+ (p)ppGpp positive control ptac::*relA* (pALS10) were also provided by D. Nguyen and obtained as previously described [32], [33]. For the expression of *relA* in the *P. aeruginosa* mutant strain PAO1Δ*relAspoT*, the pHERD20T plasmid carrying an arabinose-inducible promoter was used [34]. A 3.2 kb DNA fragment containing the *relA* gene was amplified with primers relAF (5'-GCTAGGATGCCTGCGTAATC-3') and relAR (5'-GAGATCGCCATCGAGGAATA-3') and cloned into a TOPO Zero-Blunt cloning vector (Invitrogen) and then into the pHERD20T vector. This construct was then electroporated into electrocompetent *P. aeruginosa* PAO1Δ*relAspoT* cells. Positives clones carrying the plasmid were selected on LB plus 500 µg/ml of carbenicillin, and *relA* overexpression upon induction was confirmed by RT-qPCR. In all experiments 0.01% arabinose was used to induce the promoter.

### Peptide synthesis

Peptide 1018 (VRLIVAVRIWRR-NH_2_) used in this study was synthesized by CPC Scientific using solid-phase 9-fluorenylmethoxy carbonyl (Fmoc) chemistry and purified to a purity of ∼95% using reverse-phase high-performance liquid chromatography (HPLC). Peptide mass was confirmed by mass spectrometry.

### Growth conditions

The medium used was generally BM2 minimal medium (62 mM potassium phosphate buffer, pH 7.0, 7 mM [(NH_4_)_2_SO_4_, 2 mM MgSO_4_, 10 µM FeSO_4_] containing 0.4% (wt/vol) glucose as a carbon source, except for *Staphylococcus aureus* HG001 wild-type for which BM2 glucose+0.5% casamino acids (CAA) was used, and *Salmonella enterica* SL1344 that was grown in Luria Broth. *Escherichia coli* MG1655 was grown in BM2+0.1% CAA.

### Minimal Inhibitory Concentration (MIC, MBIC_50_, MBIC_100_) assays

The broth microdilution method with minor modifications for cationic peptides [35] was used for measuring the MIC of peptide 1018. Minimal biofilm inhibitory concentrations leading to 50% decrease in biofilm growth (MBIC_50_) were obtained using 96-well plate assays and crystal violet staining of adherent biofilms as previously described [14]. The minimal peptide concentrations that completely inhibited biofilm formation (MBIC_100_) were obtained using flow cells at different input concentrations of peptide.

### Biofilm cultivation in flow cell chambers and microscopy

Biofilms were grown in flow chambers with channel dimensions of 1×4×40 mm, as previously described for 72 h at 37°C [14] in the absence or presence of the desired concentration of peptide 1018. Flow cell chambers were inoculated by injecting 400 µl of an overnight culture diluted to an OD_600_ of 0.05. After inoculation, chambers were left without flow for 2 h to enable initial adherence, after which the medium (with or without sub-inhibitory concentrations of 1018) was pumped through the system at a constant rate of 2.4 ml/h. In all cases, after 3 days of growth the flow rate (90 rpm) was increased so as to limit the amount of planktonic and loosely-attached cells within the flow cell chamber. All media used (see above) in flow cell assays supported the planktonic growth of the bacterial species tested, as determined by growth curves (e.g. Fig. S1A). Except where otherwise specified, the concentrations of peptide 1018 used were 10 µg/ml for *Pseudomonas aeruginosa*, *Escherichia coli* 0157, *Acinetobacter baumannii* SENTRY C8 and *Burkholderia cenocepacia* genomovar IIIa 4813, 20 µg/ml for *Salmonella enterica* sv. Typhimurium 14028S experiments, 2 µg/ml for *Klebsiella pneumoniae* ATTC13883, and 2.5 µg/ml for methicillin resistant *Staphylococcus aureus* MRSA #SAP0017. The different concentrations used correspond to the MBIC_100_ of the peptide against the different bacterial species as shown in Table 1. For inhibition studies, peptide was added to the flow-through medium immediately after the initial adherence phase, and maintained for 3 days. For treatment of existing biofilms, bacteria were allowed to develop into structured 2-day old biofilms prior to peptide treatment by addition into the flow cell flow-through medium for the following 24 h. Biofilm cells were stained using the LIVE/DEAD BacLight Bacterial Viability kit (Molecular Probes, Eugene, OR) or Syto-9 alone prior to microscopy experiments. A ratio of Syto-9 (green fluorescence, live cells) to propidium iodide (PI) (red fluorescence, dead cells) of 1∶5 was used. Microscopy was done using a confocal laser scanning microscope (Olympus, Fluoview FV1000) and three-dimensional reconstructions were generated using the Imaris software package (Bitplane AG). Quantification of the overall biofilm biovolume (µm^3^) and the percentage of live and dead cell volume were performed using Imaris software as previously described [7], [36]. Experiments were performed at least in triplicate.

In experiments looking at the effect of enhanced (p)ppGpp production of biofilm susceptibility to peptides, *E. coli* strain MG1655 expressing *relA* from a *lac* promoter on plasmid pALSl0 [32] was grown in flow cells for 3 days in BM2+0.1% CAA containing 20 µg/ml of 1018 and in the presence or absence of 100 µM isopropyl β-D-1-thiogalactopyranoside (IPTG). *P. aeruginosa* PAO1 and *S. aureus* HG001 strains were grown as described above but in the presence or absence of serine hydroxamate (SHX).

### Dispersal biofilm cell assay

Cell counts of live dispersed bacteria from flow cell biofilms were performed using strain *P. aeruginosa* PA14 grown in BM2 minimal glucose medium. *P. aeruginosa* PA14 biofilms were grown in the flow cell system for 2 days as described above and treated with 0.8 µg/ml or 10 µg/ml of peptide 1018. To count the dispersed viable cells, 1.5 ml of the output flow was collected at the designated times (time 0 and after 3 and 23 h) and serially diluted 10-fold. One hundred-µl portions from these serial dilutions were then plated onto LB agar plates. Plates were incubated at 37°C overnight, and colony counts were performed to obtain total CFU/ml at each time point.

### (p)ppGpp measurement by thin layer chromatography

Bacteria were grown overnight in modified MOPS minimal medium containing 0.4% glucose, 2 mM phosphate (KH_2_PO_4_), and 0.2% CAA. The cells were then diluted 1∶20 in the same MOPS minimal medium except containing 0.4 mM phosphate (KH_2_PO_4_) and 500 µM SHX to induce (p)ppGpp synthesis, in the presence or absence of peptide 1018, colistin, tobramycin or polymyxin B and cells were labelled with 10 µCi/ml ^32^P for 3 h. For experiments evaluating the ability of the peptide (or different antibiotics) to directly lead to degradation of (p)ppGpp, the cells were induced with SHX and allowed to synthesize (p)ppGpp (for 3 h) prior to peptide treatment. Samples were then extracted with frozen 13 M formic acid by three cycles of freeze-thaw. Aliquots (7.5 µl) of the supernatants were applied to 20×20 cm PEI cellulose TLC plates, resolved with 1.5 M KH_2_PO_4_ (pH 3.4) for 4 h. After chromatography, nucleotides were visualized by autoradiography and quantified with a MolecularImager FX PhosphorImager and Quantity One software (Bio-Rad). Unlabeled GTP was spotted on the plates as markers and visualized after chromatography by UV light-induced fluorescence.

### Nucleotide co-precipitation

The ability of 1018 to co-precipitate with nucleotides with varying phosphate content was examined as described previously by Hilpert et al. [37]. Peptide 1018 was mixed separately with ppGpp, GTP, GDP, ATP, ADP or NaH_2_PO_4_ in 50 mM Tris buffer at pH 7.4. ppGpp was purchased from TriLink BioTechnologies and all other nucleotides were purchased from Sigma. Nucleotide concentrations ranging from 0.25 to 0.008 mM were mixed in a microtiter plate with 0.25 nmoles of 1018 in a final volume of 100 µl per well. Samples were also prepared containing only 1018 or the nucleotide of interest at each concentration. The co-precipitation of 1018 with a nucleotide resulted in an increase in turbidity, which was quantified by measuring the A_620_ using a Powerwave X 340 microplate reader (BioTek Instruments Inc., Winooski, VT, USA).

### ^31^P-NMR spectroscopy

^31^P-NMR spectroscopy was used to evaluate the binding of 1018 to ppGpp and GTP. Samples containing 0.5 mM GTP or ppGpp were prepared in buffer (10 mM Tris, 50 mM NaCl, pH 7.4) and 1 mM phosphate was added as an internal chemical shift reference as well as for quantification. Separate samples containing 0.5 mM 1018 mixed with each nucleotide were prepared in the same way. Additional samples of 0.5 mM ppGpp, GTP and ATP with 1 mM phosphate were also prepared in 6.5 M formic acid for comparison to the samples prepared from the *P. aeruginosa* PAO1 extracts (see below). All samples contained 10% D_2_O. Each NMR sample was briefly centrifuged on a benchtop centrifuge (∼30 s) to pellet any precipitate that formed and the supernatant liquid was used as the NMR sample. ^31^P spectra were acquired at 25 °C on a Bruker Avance 500 MHz spectrometer, operating at a ^31^P frequency of 202.272 MHz. A single pulse experiment, with a 90 degree pulse of 20 µs was used, on a BB 500 probe. 4096 scans were acquired for the pure nucleotide samples while 12288 scans were accumulated for samples that contained peptide. Spectra were processed with an exponential window and line broadening of 50 Hz. To evaluate the differential binding of 1018 to ppGpp or GTP, samples containing an equimolar mixture of ppGpp and GTP (both at 0.5 mM) were prepared in Tris buffer. Peptide 1018 was added to separate nucleotide mixtures to achieve final peptide concentrations of 0.25, 0.5, 0.75 and 1 mM. The samples were again centrifuged to pellet the precipitate and the resulting supernatant was used in the NMR experiments. The experiments were performed as above, but the spectra were processed with a shifted sine bell window only. The phosphorous peak signal intensity resulting from unique chemical shift peaks from either ppGpp or GTP was determined at every concentration of 1018 tested.

To examine the effect of 1018 on ppGpp levels *in vivo*, 3×20 ml cultures of PAO1 (in BM2 media with 0.5% casamino acids) were grown overnight at 37°C in the presence of SHX (500 µM) to induce the production of ppGpp. Following overnight incubation, 1018 was added to a final concentration of 20 µg/ml and the sample was grown for an additional hour at 37°C. For comparison, a separate culture was prepared with no 1018 added. The PAO1 cells were harvested by centrifugation for 20 min at ∼2000×g in a Beckman Coulter Allegra 6 centrifuge. All three bacterial pellets were resuspended in a total of 400 µl H_2_O. To prepare the NMR sample, 400 µl of the bacteria suspension was added to 500 µl of 13 M formic acid and 100 µl of D_2_O. The sample was subjected to three rounds of freezing and thawing using liquid nitrogen and a room temperature water bath. The sample was centrifuged at 4°C and 14000 rpm in a microcentrifuge and 500 µl of the resulting supernatant was used as the NMR sample. Spectra were acquired as described for the pure nucleotide samples but with an accumulation of 24576 scans.

## Supporting Information

Figure S1 (**A**) **(p)ppGpp mutants exhibited reduced ability to form biofilms in flow cells.** Biofilms were grown in flow cells and subsequently imaged and analyzed as outlined in the Methods section. Briefly, bacteria were stained with the all-bacteria Syto-9 stain and analyzed using confocal microscopy. Three-dimensional biofilm reconstructions were generated using Imaris software. (p)ppGpp mutants of the different bacterial species showed decreased biofilm formation in flow cells compared to their parent strains (left panel) using media that supported planktonic growth of both parent and mutant strains in each case (right panel). For assessing planktonic growth, cells were grown in 96-well microtiter plates and growth assessed at 37°C under shaking conditions using a TECAN Spectrofluor Plus. The medium used was BM2 minimal medium glucose for *P. aeruginosa* Δ*relAspoT* and its parent strain. BM2+0.1% CAA was used in the case of *Escherichia coli* MG1655 and its mutant. LB medium was used for *Salmonella enterica* SL1344 and its mutant. BM2 glucose+0.5% casamino acids was used to grow *Staphylococcus aureus* HG001 wild-type and its *rsh* mutant. (**B**) Mutations in both genes responsible for (p)ppGpp synthesis as well as treatment with modest amounts (0.8 µg/ml) of peptide 1018 caused filamentation and cell death (as revealed by the uptake of propidium iodide that stains bacteria red) of bacteria grown under biofilm conditions in flow cells. (**C**) **Effect of increasing SHX levels on PAO1 planktonic growth.** *P. aeruginosa* PAO1 was grown in BM2 minimal medium and exposed to increasing concentrations of SHX. The growth of these cultures at 37°C under shaking conditions was monitored with a TECAN Spectrofluor Plus by determining the absorbance at 620 nm for 24 h.
(TIF)

Figure S2 **Arabinose did not affect biofilm formation or viable cell dispersal from flow cell biofilms.** (**A**) Addition of 0.01% arabinose to flow-through medium for 3 days or for only the first 2 days of the experiment (conditions identical to those of Fig. 5) did not alter biofilm formation in *P. aeruginosa* PAO1. Bacteria were stained with the all-bacteria Syto-9 stain and analyzed using confocal microscopy. Three-dimensional biofilm reconstructions were generated using Imaris software. Four independent experiments were performed. (**B**) Exogenous addition of 0.01% arabinose for 3 days or 2 out of 3 days (as in Fig. 5) did not increase cell dispersal from biofilms. Dispersed cells from 2-day old biofilms were collected and viable cell counts performed 3 and 6 h after addition of arabinose was either discontinued for the last 24 h of the experiment or not. Four independent experiments were performed. Student's *t* test was used (ns, P>0.05).
(TIF)

Figure S3 **Conventional antibiotics did not prevent (p)ppGpp accumulation and did not degrade (p)ppGpp.** To evaluate whether antibiotics inhibited (p)ppGpp formation they were added at the same time as SHX and (p)ppGpp pools were observed after 3 h using TLC (left panel). All antibiotics were unable to prevent ppGpp accumulation in fact both tobramycin and polymyxin B increased ppGpp levels (left panel). For degradation experiments, (p)ppGpp levels were allowed to increase by growth of cells for 3 h in the presence of SHX as described in Methods and cells were then treated with the different antibiotics. After 5 and 30 min, levels of (p)ppGpp were determined using TLC. Treatment with colistin and polymyxin B for 30 minutes did not lead to direct degradation of (p)ppGpp (right panel). Accumulation of ppGpp upon polymyxin B treatment was more substantial in the inhibition experiments (left panel) than in the degradation experiments (right panel).
(TIF)

Figure S4 **Addition of serine hydroxamate stimulated (p)ppGpp accumulation, which was blocked by treatment with peptide 1018.** (**A**) **SHX addition led to (p)ppGpp accumulation in planktonic cells.** *P. aeruginosa* PAO1 was grown overnight in MOPS minimal medium containing 0.4% glucose, 2 mM phosphate (KH_2_PO_4_), and 0.2% CAA. The cells were then diluted 1∶20 in the same MOPS minimal medium except containing 0.4 mM phosphate (KH_2_PO_4_). Cells were labelled with 10 µCi/ml ^32^P for 3 h. (p)ppGpp synthesis was induced with 500 µM serine hydroxamate (SHX). (**B**) **Peptide 1018 blocked (p)ppGpp accumulation in** ***Acinetobacter baumannii*** **and** ***Klebsiella pneumoniae*** **.** Anti-biofilm peptide 1018 at 5 µg/ml directly prevented (p)ppGpp accumulation in strains *A. baumannii* SENTRY C8 and *K. pneumoniae* ATTC13883 as revealed by thin layer chromatography separation of guanine nucleotides extracted from intact cells. Results shown were obtained using thin layer chromatography and correspond to samples untreated and treated with peptide 1018.
(TIF)

Figure S5 **^31^P-NMR studies showed that peptide 1018 preferentially bound to ppGpp compared to GTP.** (**A**) **Binding of ppGpp and GTP by the anti-biofilm peptide 1018 monitored with ^31^P-NMR.** ^31^P-NMR spectra were acquired for 0.5 mM samples of GTP or ppGpp in 10 mM Tris pH 7.4 or in 6.5 M Formic Acid (Top panel). Separate samples were prepared containing 0.5 mM nucleotide and 0.5 mM 1018 (Bottom panel). The samples were centrifuged and the supernatant was collected and used as the NMR sample. For the samples prepared in Tris buffer, 1018 precipitated GTP and ppGpp from solution resulting in a significant decrease in the amount of free nucleotide remaining in solution and a large reduction in the phosphorous signals in the ^31^P NMR spectra (bottom panel). In contrast, no precipitate was observed between ppGpp and 1018 under acidic conditions (6.5 M formic acid). The peaks arising from the nucleotide of interest are indicated while the unlabelled peak corresponds to the 1 mM NaH_2_PO_4_ added to the sample as an internal standard. (**B**) **Effect of increasing amounts of 1018 on the ^31^P-NMR signal intensities arising from ppGpp and GTP in NMR samples containing an equimolar mixture of both nucleotides (0.5 mM each).** Peak intensities were measured as a relative value compared to the internal reference peak of 1 mM phosphate at ∼4 ppm. The preferential precipitation of ppGpp over GTP by 1018 is evident from the larger decrease in ppGpp phosphorous signals (at −4.2 and −5.5 ppm) compared to the GTP signals (at −5 and −20 ppm). It should be noted that the ppGpp and GTP phosphorous signals at approximately 9 ppm overlapped with one another and could therefore not be examined in this manner.
(TIF)

Figure S6 **Determination of ^31^P chemical shifts for ppGpp, GTP and ATP under nucleotide extraction conditions and monitoring ppGpp levels** ***in vivo*** **from** ***P. aeruginosa*** **PAO1 cell extracts.** NMR spectra of 0.5 mM ppGpp, GTP or ATP in 6.5 M formic acid were collected as described in Figure S5. Each sample also contained 1 mM phosphate as an internal chemical shift reference (peak at ∼1.5 ppm). These spectra were used to establish the chemical shifts of these compounds under the acidic conditions employed to extract the nucleotides from PAO1 cultures. The complete ^31^P-NMR spectrum of nucleotides extracted from PAO1 cultures induced with SHX is shown in the bottom panel. The ppGpp spectrum had a unique peak at ∼0.6 ppm which did not overlap with phosphorous peaks from either GTP or ATP (indicated with an arrow and a vertical dashed line). This peak was used to evaluate the ppGpp levels in the nucleotides extracted from bacterial cultures.
(TIF)
