# Supplementary figures and images for: Broad-Spectrum Anti-biofilm Peptide That Targets a Cellular Stress Response
Source: PLoS Pathog. 2014 May 22;10(5):e1004152. doi: 10.1371/journal.ppat.1004152 (PMC4031209; doi:10.1371/journal.ppat.1004152)

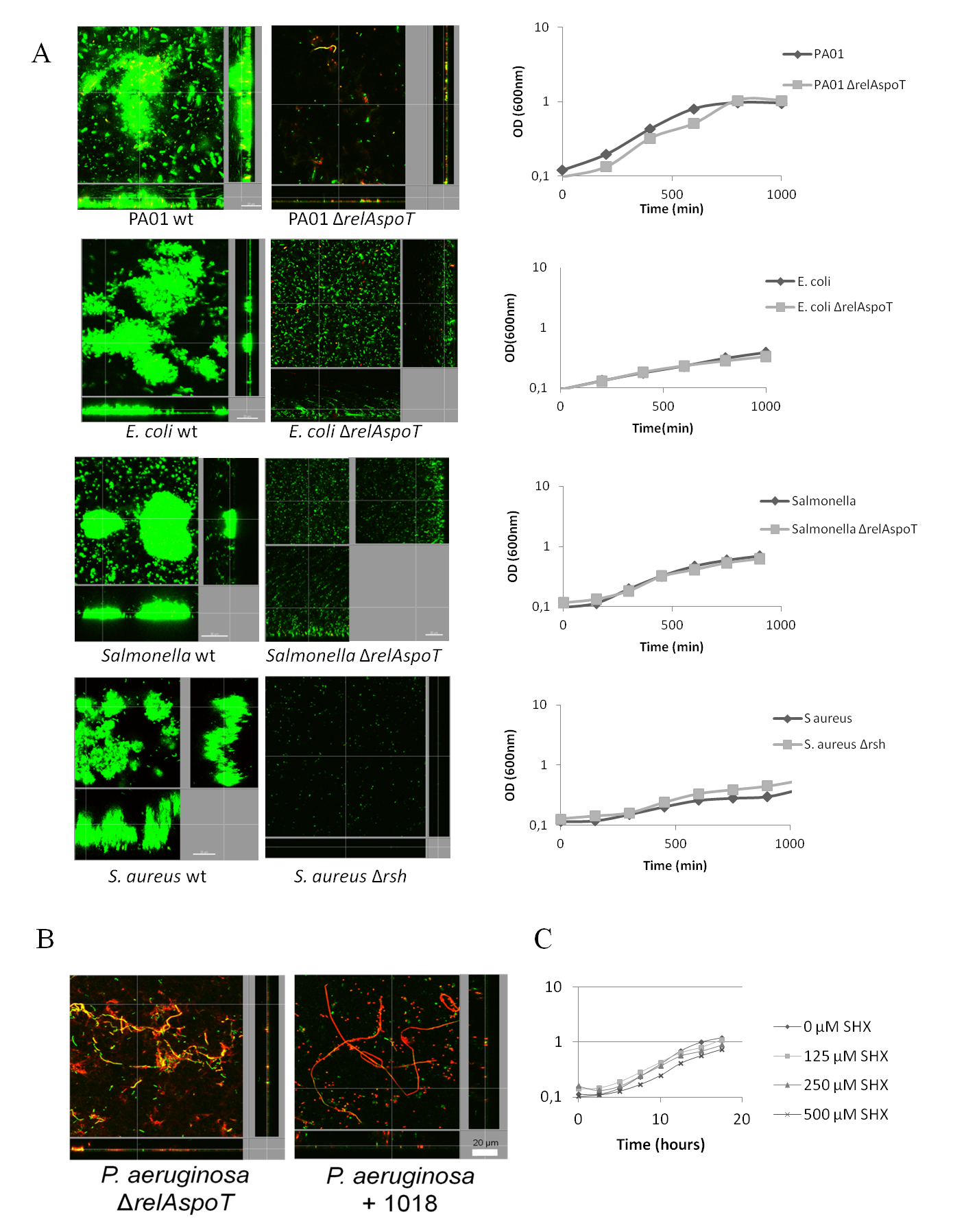

Supplement: Figure S1 — (A) (p)ppGpp mutants exhibited reduced ability to form biofilms in flow cells. Biofilms were grown in flow cells and subsequently imaged and analyzed as outlined in the Methods section. Briefly, bacteria were stained with the all-bacteria Syto-9 stain and analyzed using confocal microscopy. Three-dimensional biofilm reconstructions were generated using Imaris software. (p)ppGpp mutants of the different bacterial species showed decreased biofilm formation in flow cells compared to their parent strains (left panel) using media that supported planktonic growth of both parent and mutant strains in each case (right panel). For assessing planktonic growth, cells were grown in 96-well microtiter plates and growth assessed at 37°C under shaking conditions using a TECAN Spectrofluor Plus. The medium used was BM2 minimal medium glucose for P. aeruginosa ΔrelAspoT and its parent strain. BM2+0.1% CAA was used in the case of Escherichia coli MG1655 and its mutant. LB medium was used for Salmonella enterica SL1344 and its mutant. BM2 glucose+0.5% casamino acids was used to grow Staphylococcus aureus HG001 wild-type and its rsh mutant. (B) Mutations in both genes responsible for (p)ppGpp synthesis as well as treatment with modest amounts (0.8 µg/ml) of peptide 1018 caused filamentation and cell death (as revealed by the uptake of propidium iodide that stains bacteria red) of bacteria grown under biofilm conditions in flow cells. (C) Effect of increasing SHX levels on PAO1 planktonic growth. P. aeruginosa PAO1 was grown in BM2 minimal medium and exposed to increasing concentrations of SHX. The growth of these cultures at 37°C under shaking conditions was monitored with a TECAN Spectrofluor Plus by determining the absorbance at 620 nm for 24 h. (TIF) [file ppat.1004152.s001.tif]

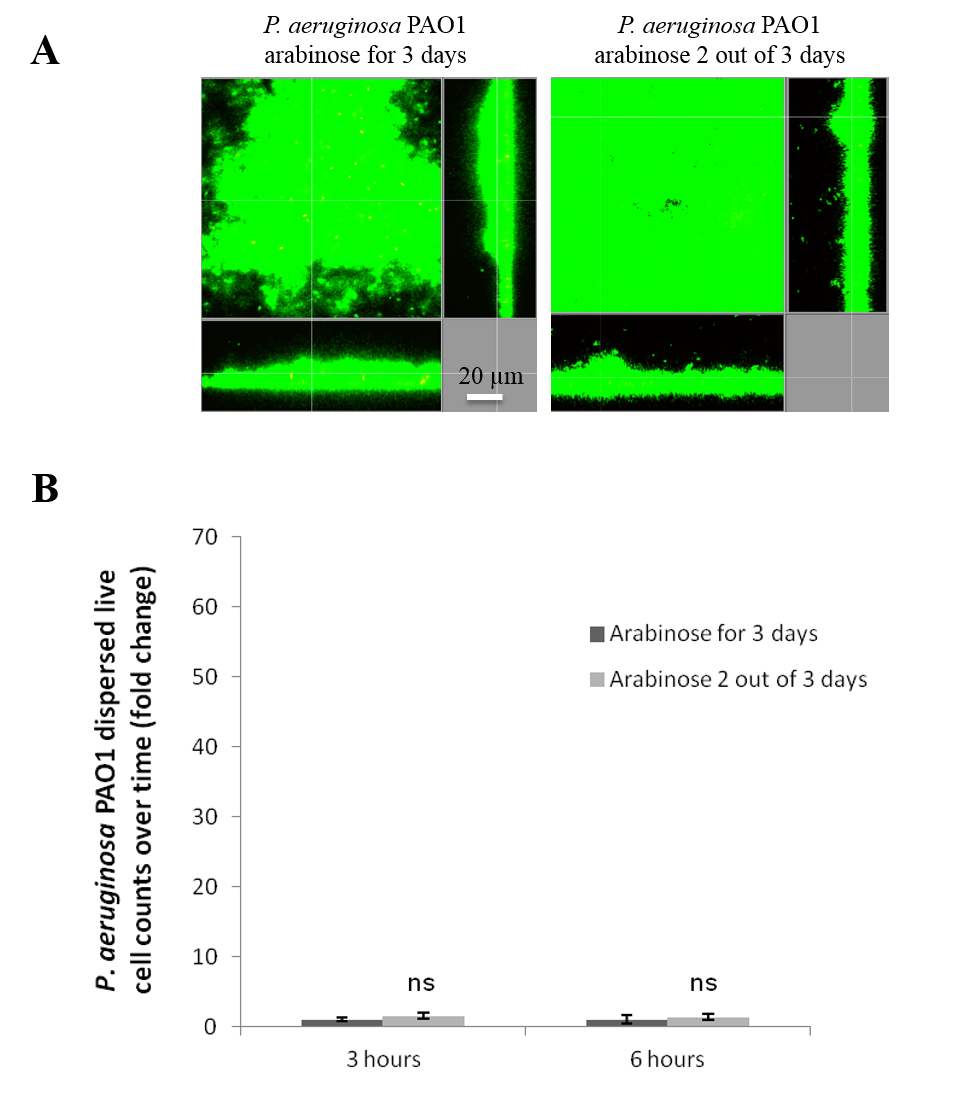

Supplement: Figure S2 — Arabinose did not affect biofilm formation or viable cell dispersal from flow cell biofilms. (A) Addition of 0.01% arabinose to flow-through medium for 3 days or for only the first 2 days of the experiment (conditions identical to those of Fig. 5) did not alter biofilm formation in P. aeruginosa PAO1. Bacteria were stained with the all-bacteria Syto-9 stain and analyzed using confocal microscopy. Three-dimensional biofilm reconstructions were generated using Imaris software. Four independent experiments were performed. (B) Exogenous addition of 0.01% arabinose for 3 days or 2 out of 3 days (as in Fig. 5) did not increase cell dispersal from biofilms. Dispersed cells from 2-day old biofilms were collected and viable cell counts performed 3 and 6 h after addition of arabinose was either discontinued for the last 24 h of the experiment or not. Four independent experiments were performed. Student's t test was used (ns, P>0.05). (TIF) [file ppat.1004152.s002.tif]

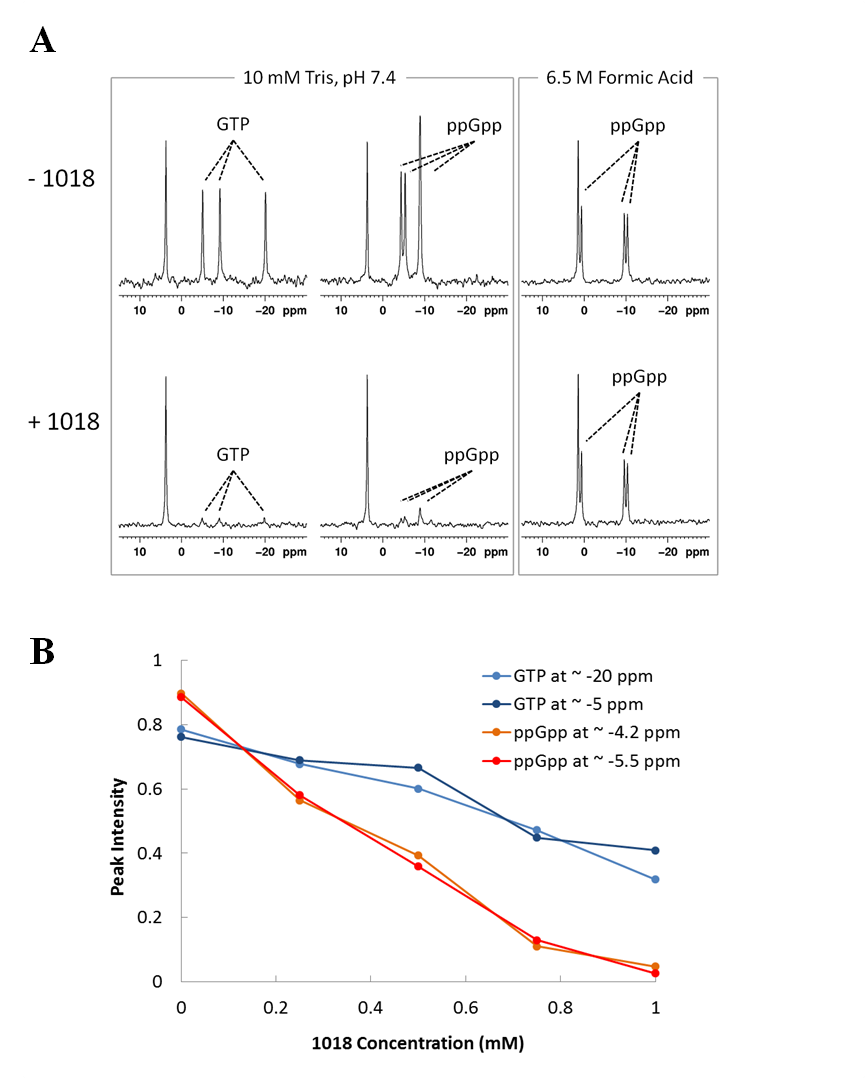

Supplement: Figure S5 — 31P-NMR studies showed that peptide 1018 preferentially bound to ppGpp compared to GTP. (A) Binding of ppGpp and GTP by the anti-biofilm peptide 1018 monitored with 31P-NMR. 31P-NMR spectra were acquired for 0.5 mM samples of GTP or ppGpp in 10 mM Tris pH 7.4 or in 6.5 M Formic Acid (Top panel). Separate samples were prepared containing 0.5 mM nucleotide and 0.5 mM 1018 (Bottom panel). The samples were centrifuged and the supernatant was collected and used as the NMR sample. For the samples prepared in Tris buffer, 1018 precipitated GTP and ppGpp from solution resulting in a significant decrease in the amount of free nucleotide remaining in solution and a large reduction in the phosphorous signals in the 31P NMR spectra (bottom panel). In contrast, no precipitate was observed between ppGpp and 1018 under acidic conditions (6.5 M formic acid). The peaks arising from the nucleotide of interest are indicated while the unlabelled peak corresponds to the 1 mM NaH2PO4 added to the sample as an internal standard. (B) Effect of increasing amounts of 1018 on the 31P-NMR signal intensities arising from ppGpp and GTP in NMR samples containing an equimolar mixture of both nucleotides (0.5 mM each). Peak intensities were measured as a relative value compared to the internal reference peak of 1 mM phosphate at ∼4 ppm. The preferential precipitation of ppGpp over GTP by 1018 is evident from the larger decrease in ppGpp phosphorous signals (at −4.2 and −5.5 ppm) compared to the GTP signals (at −5 and −20 ppm). It should be noted that the ppGpp and GTP phosphorous signals at approximately 9 ppm overlapped with one another and could therefore not be examined in this manner. (TIF) [file ppat.1004152.s005.tif]
